# Effects of Total Alkaloids of* Sophora alopecuroides* on Biofilm Formation in* Staphylococcus epidermidis*

**DOI:** 10.1155/2016/4020715

**Published:** 2016-06-19

**Authors:** Xue Li, Cuiping Guan, Yulong He, Yujiong Wang, Xiaoming Liu, Xuezhang Zhou

**Affiliations:** ^1^Key Laboratory of the Ministry of Education for the Conservation and Utilization of Special Biological Resources of Western China, Yinchuan, Ningxia 750021, China; ^2^College of Life Science, Ningxia University, Yinchuan, Ningxia 750021, China; ^3^Ningxia Key Laboratory of Clinical and Pathogenic Microbiology, General Hospital of Ningxia Medical University, Yinchuan, Ningxia 750004, China

## Abstract

*Staphylococcus epidermidis* (*S. epidermidis*) is an opportunistic pathogen with low pathogenicity and a cause of the repeated outbreak of bovine mastitis in veterinary clinical settings. In this report, a biofilm model of* S. epidermidis* was generated and the minimal inhibitory concentration (MIC) and sub-MIC (SMIC) on bacterial cultures were assessed for the following agents: total alkaloids of* Sophora alopecuroides* (TASA), ciprofloxacin (CIP), and erythromycin (ERY). The formation and characteristic parameters of biofilm were analyzed in terms of XTT assay, silver staining, and confocal laser scanning microscope (CLSM). Results showed that a sub-MIC of TASA could inhibit 50% biofilm of bacterial activity, while 250-fold MIC of CIP and ERY MICs only inhibited 50% and 47% of biofilm formation, respectively. All three agents could inhibit the biofilm formation at an early stage, but TASA showed a better inhibitory effect on the late stage of biofilm thickening. A morphological analysis using CLSM further confirmed the destruction of biofilm by these agents. These results thus suggest that TASA has an inhibitory effect on biofilm formation of clinic* S. epidermidis*, which may be a potential agent warranted for further study on the treatment prevention of infection related to* S. epidermidis* in veterinary clinic.

## 1. Introduction

*Staphylococcus epidermidis* (*S. epidermidis*) is a natural colonizer on healthy human skin and mucosa, which is one example of coagulase-negative staphylococci (CNS) [1–3].* S. epidermidis* is generally considered as a nonpathogenic bacterium, but mounting evidences have recently suggested that it is also a main pathogenic bacterium responsible for many indwelling medical device related infections, including chronic prostatitis, septicemia, and mastitis, owing to its ability to create biofilm on biomaterial surfaces [4]. Biofilms of* S. epidermidis* are comprised of clusters of cells encased in a self-synthesized extracellular polymeric matrix. The biofilm matrix can hold the cells together, attach them to the surface, and protect them from killing by host defenses and antimicrobial agents [5]. Biofilm bacteria are thus generally more resistant to antibiotics and host immune attack in comparison with planktonic cells for several reasons [6]. First, the biofilm forms a protective physical barrier that protects biofilm bacteria by limiting contact with antimicrobial agents; secondly, the bacterial cells inside of the biofilm are in a state of low energy, low activity, and low metabolism; third, antimicrobial related genes can be horizontally transferred or mutated when bacteria are stimulated by an external stress and accordingly alter the gene expression profile and transmit the antibiotic resistance to sensitive strains; finally,* S. epidermidis* can produce persister cells in response to an antibiotic stress. Indeed, a drug resistance of bacteria has been recognized to relate to persister cells at some extent, as targeted enzymes of antibiotics may be inactivated in persister cells of biofilm, which could lead bacteria to survive under an antibiotic action [7]. Therefore the resistance of biofilm bacteria to antibiotics is higher than that of floating ones [8, 9].

Mastitis is a global problem that causes huge economic losses in dairy industries, due to poor milk quality, reduced milk yield, and increased expenditure on treatment and even animal death caused by the disease itself or through culling of affected cows [10]. In China, bovine mastitis is a serious problem in dairy industries and the average incidence rate is up to 33% [11].* S. epidermidis* and other coagulase-negative staphylococci are considered to be major mastitis pathogens. Antimicrobial treatment is often used to decrease the incidence or shorten the duration of bovine mastitis. However, a failure of treatment occurs owing to the development of antibiotic resistance [12]. With an emergence of antibiotic-resistant bacterial isolates found in the mastitis of dairy livestock as well as the increasing evidence of* S. epidermidis* relevant mastitis pathogen [12, 13], it is necessary to discover or develop novel agents or regimens for the prevention and treatment of mastitis in dairy animals.

Traditional Chinese medicine (TCM) has been used in China over thousands of years for the prevention and treatment of various diseases, including infectious diseases. Recently, TCM has spurred increased interests owing to its potential therapeutic effects and minimal side effects [14]. Therefore, it might be of importance in exploiting Chinese herbs with antimicrobial activity to address problems caused by the development of drug-resistant strains and shortage of effective antibiotics for drug-resistant bacteria infections.* Sophora alopecuroides* is a plant from a genus of Leguminosae family; it mainly grows in the desolate salt marshes or saline-alkali lands in desert region of Northwest China and North China, as well as in the Central Asian region [15]. The main active components in Sophora plants are quinolizidine alkaloids, such as the total alkaloids of* Sophora alopecuroides* (TASA). TASA have been proved to have activities of anti-inflammation and antibacteria, as well as antioxidant, immunomodulatory, and anticancer functions. With respect to its antimicrobial activities, we suggest that TASA may be a promising agent for the prevention and treatment of mastitis in animal practice when it is used alone or in combination with commonly used antibiotics [16]. Together with the evidence of TASA capable of reversing the resistance of* E. coli* to antibiotics [17] and the safety of TASA in clinical application with lower toxicity [18], we thus hypothesized that the TASA may also have an activity to inhibit biofilm bacteria of* S. epidermidis*. In the present study, we therefore compared TASA with erythromycin and ciprofloxacin to seek potential effect of eliminating biofilm of* S. epidermidis* using TASA.

## 2. Materials and Methods

### 2.1. Bacterial Strains and Medicines

*S. epidermidis* strain ATCC12228 was purchased from Guangzhou Microbiological Culture Collection Center (Guangzhou, China);* S. epidermidis* strain ATCC35984 was kindly provided by professor Guan Yan in Anhui University of Chinese Medicine (Hefei, China);* S. epidermidis* isolate 30 was a clinical stain isolated from milk samples produced from a cow with mastitis, which was identified by biochemical characterizations and genetic confirmation by a polymerase chain reaction assay with species-specific primer sets against* S. epidermidis* [19]. Total Alkaloids of* Sophora alopecuroides* (TASA, total alkaloids 95%) were obtained from the Redbud Pharmaceutical Factory (Yinchuan, Ningxia, China, Batch number 9600169). Erythromycin (ERY) and ciprofloxacin (CIP) were purchased from Pharmaceutical and Biological Products Inc. (Beijing, China).

### 2.2. Media and Chemicals

Chemicals used in this study were products of Sigma (St. Louis, MO, USA), unless otherwise indicated. Muller-Hinton (MH) agar and broth were OXOID products (UK). DNA fluorescent dye L13152 LIVE/DEAD BacLight Bacterial Viability Kits (Molecular Probes Co., Ltd., USA); XTT cell proliferation; and cytotoxicity test kit were purchased from Nanjing KeyGen Biotech. Co., Ltd. (Nanjing, China), E.Z.N.A.*™* Bacterial RNA Kit was a product of Omega Bio-Tek (Norcross, GA, USA).

### 2.3. Minimum Inhibitory Concentration Test

Antibacterial activity was evaluated by accessing the minimum inhibitory concentration (MIC), using a broth dilution assay. The effects of antimicrobial agents TASA, ERY, and CIP on bacterium strains of* S. epidermidis* isolate 30, ATCC12228, and ATCC35984 were examined. Briefly, the bacterial culture was subcultured on MH agar at 37°C overnight. Single colonies were picked and inoculated into MH broth. The inoculum was adjusted to 1 × 10^8^ colony-forming units (CFU)/mL by comparison with a 0.5 McFarland turbidity standard. 100 *μ*L bacterial suspension and 100 *μ*L of the tested agent at different concentration were then mixed in a well of a 96-well cell culture plate. The concentrations of tested agents were ranged from 32 to 0.125 *μ*g/mL for ERY and CIP and 50 to 0.15625 mg/mL for TASA. The minimum inhibitory concentration (MIC) is defined as the minimum concentration of an agent that completely inhibited the visible growth of microorganisms, which was determined in a cultivation that has been cultured at 37°C for 18 h. The experiments were repeated for a minimum of 3 times.

### 2.4. Effect of TASA, ERY, and CIP on Biofilm

*S. epidermidis* isolate 30 was routinely grown in Tryptic Soy Broth (TSB) at 37°C with shake overnight. 100 *μ*L of precultured suspension of a test strain at a density of 1 × 10^6^ CFU/mL in TSB was added to each well of 96-well cell culture plate, and the culture was incubated at 37°C for an additional 24 h. The well was then washed three times with PBS, followed by an addition of twofold serial dilutions of tested agent into the well. For TASA, starting concentration was 20 mg/mL and final diluted concentration was 0.625 mg/mL, and for ERY and CIP, starting concentration was 1000 *μ*g/mL and final diluted concentration was 31.25 *μ*g/mL. These concentrations were chosen according to a previous study reported by Guan et al. [20]. Each drug concentration was performed for 6 repeats, and the TSB was used as negative control. The cultures were incubated at 37°C for 24 h, then the culture medium was discarded, and the well was washed with PBS for three times. After that, 200 *μ*L of 0.5% crystal violet was added to the well and incubated at room temperature for 20 min; then running water was used for washing away the excessive crystal violet. After several hours of drying at room temperature, 200 *μ*L absolute ethyl alcohol was added and absorbance at OD_570_ was measured. This experiment was repeated for 3 times, and the results were recorded. SMIC_80_ represented a concentration of tested agent that could inhibit 80% biofilm bacteria compared to negative control and the negative control was regarded as 100%. Similar to SMIC_80_, SMIC_50_ means inhibiting 50% biofilm bacteria.

### 2.5. Effects of TASA, ERY, and CIP on the Biofilm Formation Process of* S. epidermidis* Isolate 30

The clinical* S. epidermidis* isolate 30 was cultured for 18 h prior to being diluted in broth at a density of 1 × 10^6^ CFU/mL. For each well of a 96-well microplate, a 200 *μ*L broth was added and cultured at 37°C. The culture medium was discarded at 0 h, 2 h, 6 h, 12 h, or 24 h after the inoculation. The well was washed with sterile water for 3 times, then ERY and CIP at a concentration of 1000, 100, and 10 mg/L and TASA at a concentration of 20, 10, and 5 mg/mL were added, and the cultures were incubated at 37°C for 24 h, respectively. These concentrations were chosen based on results of SMIC_50_ and SMIC_80_. The medium was removed and the well was washed with PBS twice. 100 *μ*L of TSB and 20 *μ*L of XTT solution were then added to each well and incubated at 37°C for additional 2 h in dark. Since the cleavage of XTT by dehydrogenase enzymes of metabolically active cells in biofilms yields a highly colored formazan product that could be photometrically measured [21, 22], XTT assay was thus employed for ascertaining the drug effect on bacterial biofilm in this study. The TSB was also set as negative control and absorbance at 450 nm was measured.

### 2.6. Fractional Inhibitory Concentration Index (FICI) Tested with Checkerboard Assay

Values of FICIs were used for evaluating antibiotic interactions between TASA, ERY, and CIP. MIC values of TASA, ERY, and CIP were tested alone. FICI was determined by MICs for each combination of two agents and was calculated by using the following equation: FICI = FICA + FICB, where FICA = MIC of drug A in combination/MIC of drug A alone and FICB = MIC of drug B in combination/MIC of drug B alone. The FICI was interpreted as follows: FICI ≤ 0.5; there was synergistic effect; 0.5 < FICI ≤ 1.0; there was additive effect; 1.0 < FICI ≤ 4.0; there was no interaction; FICI > 4.0; there was antagonistic effect [15].

### 2.7. Morphological Structure of Biofilm of the* S. epidermidis* Isolate 30 after Treatments of TASA, ERY, and CIP as Examined by a Fast Silver Staining Assay

Experimental bacterium* S. epidermidis* isolate 30 was cultured for 18 h at 37°C with shaking at 150 r/min. The broth was then diluted to 0.5 McFarland with TSB medium. An aseptic cover glass was then laid at the bottom of 6-well tissue culture plate, and 3 mL of bacterial suspension was added into each well and then ERY (500 mg/L), CIP (500 mg/L), and TASA (20 mg/mL) were added and incubated at 37°C for 24 h. These concentrations were chosen based on results of SMIC_50_ and SMIC_80_. The culture medium was discarded and the cover glass was washed 3 times with the sterilized distilled water. Then 2.5% glutaraldehyde was added to fix the biofilm for 90 min. After discarding the liquid waste, the plate was then washed 3 times with the sterilized distill water, followed by adding 1 mL of saturated CaCl_2_ and incubating for 15 min. After discarding the solution and washing the plate for 3 times, 1 mL of AgNO_3_ was added and incubated for 15 min. 1 mL benzenediol was added and incubated for 2 min after removing of THE AgNO_3_ solution, followed by discarding the benzenediol solution and washing the plate for 3 times; Na_2_S_2_O_3_ was used to fix the biofilm for 2 min before it was discarded. After a final washing of the plate for 3 times, the morphological structure of biofilm of* S. epidermidis* isolate 30 was examined with the oil immersion lens under a microscope (Hitachi Limited, Japan).

### 2.8. Effects of TASA, ERY, and CIP on the Morphological Structure of Biofilm of* S. epidermidis* Isolate 30 Examined by Confocal Laser Scanning Microscope (CLSM)

Concentrations of ERY, CIP, and TASA used in this assay were 500 *μ*g/mL, 500 *μ*g/L, and 20 mg/mL, respectively. For CLSM analysis of biofilm, above treated bacteria cultured on glass cover slides were incubated with 5 *μ*L DNA fluorescent staining solution (the fluorescent staining solution was mixed by SYTO9 and propidium iodide prior to be applied). The plate was incubated at 37°C for 15 min in dark. After washing for 3 times with PBS, the aseptic cover glass was placed on a glass slide [20]. The stained biofilm was then examined with CLSM with parameters of green exciting light at 488 nm, red exciting light at 543 nm, 40x objective, and 10x eyepiece using a Leica TCS SP2 A0BS Confocal System and processed on Leica Confocal Software v.2.6.1 (Leica, Germany). Each group was repeated three times and 3 different fields were randomly selected for each sample. Then the selected fields were scanned layer by layer from 4 to 20 layers based on the thickness of biofilm. Viable bacteria were displayed in green fluorescent, dead bacteria were imaged in red fluorescent, and an area with both viable and dead bacteria was superimposed as* aurantiacus* fluorescence in images of CLSM.

### 2.9. Quantitative Analysis of Biofilm Structure Using ISA Software

Shear layer pictures from different cultured time of biofilm were imported into the ISA software, which was powerful analytic software for biofilms [23]. The ISA&ISA3D program was used for this analysis. The running parameters were biovolume (BV), average diffusion distance (ADD), areal porosity (AP), and textual entropy (TE). These parameters could reflect the number of biofilm bacteria, the density of biofilm, and the metabolize of biofilm [23]. In this regard, AP and ADD could objectively reflect the variation of gaps and distance of nutrient transport in the structure development of biofilm, and the TE could reflect the variation of homogeneity in the biofilm formation.

### 2.10. Data Analysis

The data were analyzed with SPSS software. For two-sample comparison, Student's *t*-test was used. For comparison of groups more than two, one-way ANOVA was applied. *p* < 0.05 was considered as a statistically significant difference, while *p* < 0.01 was considered extremely significant.

## 3. Results

### 3.1. Minimum Inhibitory Concentration of TASA, ERY, and CIP on* S. epidermidis* Strains

Minimum inhibitory concentration (MIC) of* S. epidermidis* 30 isolate for TASA, ERY, and CIP was detected by broth dilution method. For* S. epidermidis* isolate 30, its MICs of TASA, ERY, and CIP were 20 mg/mL, 4 mg/L, and 0.5 mg/L, respectively. For* S. epidermidis* reference strain ATCC35984, its MICs of TASA, ERY, and CIP were 10 mg/mL, 2 *μ*g/mL, and 0.25 *μ*g/mL, respectively. For strain ATCC12228, its MICs of TASA, ERY, and CIP were 10 mg/mL, 1 *μ*g/mL, and 0.25 *μ*g/mL, respectively.

### 3.2. Effect of TASA, ERY, and CIP on* S. epidermidis* Biofilm Formation

**Figure 1:**
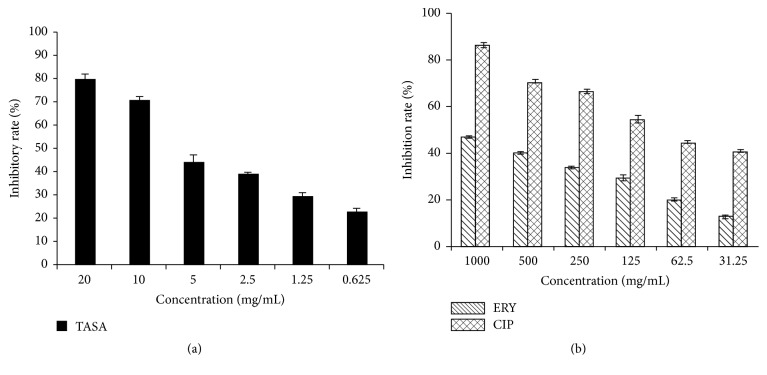
A dose-dependent effect of antimicrobial agents on biofilm formation of* S. epidermidis*. The inhibitory rates of biofilm of* S. epidermidis* isolate 30 were determined by the SMIC using a crystal violet staining assay. (a) The inhibitory rate of biofilm formation by TASA; (b) the inhibitory rate of biofilm formation by CIP and ERY.

In order to access inhibitory rates of TASA, ERY, and CIP for the biofilm formation* S. epidermidis* isolate 30, SMIC_50_ or SMIC_80_ was determined. SMIC_80_ represents the drug concentration that could inhibit 80% biofilm bacteria compared to negative control, of which the OD value of negative control was regarded as 100%. Similar to SMIC_80_, SMIC_50_ means an inhibition of 50% growth of bacterial biofilm. Results showed that SMIC_50_ of TASA was 5 mg/mL and SMIC_80_ of TASA was 20 mg/mL, as compared to the MIC of TASA. This suggested that TASA could inhibit biofilm bacteria at a concentration of sub-MIC (Figure 1(a)). Furthermore, 80% biofilm bacteria were inhibited when the CIP concentration was at 2000 times of its MIC; 54% biofilm bacteria were inhibited when its concentration was 250 times higher than its MIC. In addition, only 47% biofilm bacteria were inhibited when ERY concentration was at 250 times of its MIC (Figure 1(b)). These results indicated that once the bacteria biofilm was formed, the resistance to antibiotics was significantly increased. In this context, ERY could fully inhibit the growth planktonic bacteria of* S. epidermidis* isolate 30 at 4.0 *μ*g/mL, but it only inhibited about 50% biofilm bacteria of this isolate at 1000 *μ*g/mL, similar to CIP, which was able to completely inhibit planktonic bacteria of* S. epidermidis* isolate 30 at 0.5 *μ*g/mL but only inhibited about 50% biofilm growth of this clinical isolate at 62.5 *μ*g/mL. Furthermore, a significant dose-dependent inhibition of bacterial growth was also determined between different agents at indicated concentrations (*p* < 0.05).

### 3.3. Effects of TASA, ERY, and CIP on the Process of Biofilm Formation of* S. epidermidis* Isolate 30

**Figure 2:**
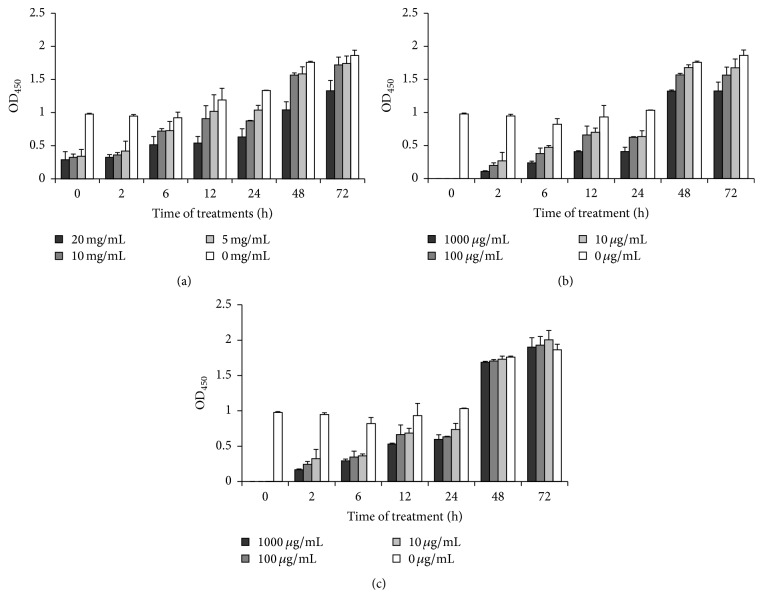
Time- and dose-dependent effects of TASA, ERY, and CIP on adhesion of* S. epidermidis*. The experiment drug effect on* S. epidermidis* isolate 30 was tested by an XTT assay. Time- and dose-dependent inhibitory effects of TASA (a), CIP (b), and ERY (c) on inhibiting* S. epidermidis* isolate 30 were analyzed.

A time- and dose-dependent inhibition of biofilm formation was found in* S. epidermidis* isolate 30 treated with different concentration of TASA for 0, 2, 6, 12, 24, 48, and 72 h after incubation, as compared with untreated controls. ERY and CIP could inhibit the biofilm formation at 0, 2, 6, 12, and 24 h when their concentrations were at 10 *μ*g/mL, 100 *μ*g/mL, and 1000 *μ*g/mL. However, the two agents had no significant inhibitory effect at 48 and 72 hours (*p* > 0.05) (Figure 2). There were differences between each growth period treated with different agents (*p* < 0.01 or *p* < 0.05). These data showed that ERY and CIP have more inhibitory effects than TASA at initial phase of biofilm formation. However, the inhibitory effects of ERY and CIP on biofilm growth waned down with times at 48 h and 72 h, and the ERY treatment even showed a promotion in biofilm formation at 72 h. In contrast, the TASA exhibited a consistent inhibitory effect on biofilm growth at 72 h of treatment (Figure 2).

### 3.4. Synergistic Antimicrobial Effects of TASA and ERY or CIP on* S. epidermidis* as Determined by FICI

The antibacterial activity of TASA combined with ERY or CIP* in vitro* on ATCC12228, ATCC35984, and* S. epidermidis* isolate 30 was listed in Tables 1 and 2. It was observed that there was no synergistic effect between TASA and ERY or CIP on above three strains. Also, there was no interaction between ERY and TASA either, when assayed on* S. epidermidis* 30. This result suggested that a combination of TASA with ERY or CIP could not enhance its antibacterial effect on biofilm-producing* S. epidermidis*.

### 3.5. Morphological Structure of Biofilm of* S. epidermidis* Isolate 30 Observed by a Fast Silver Staining Assay

**Figure 3:**
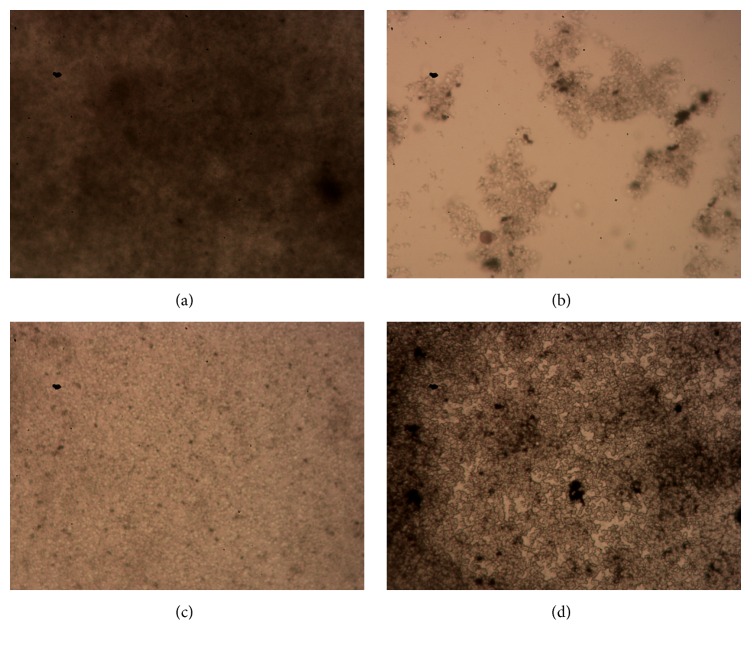
Representative images of biofilms of* S. epidermidis* isolate 30 treated with different agents. The morphological structures of BFs of* S. epidermidis* isolate 30 treated with TSB control (a), 10 mg/mL of TASA (b), 500 mg/L of CIP (c), and 500 mg/L of ERY for 24 hours were examined by a fast silver staining method.

Compared with the controls (Figure 3(a)), all biofilms from drug-treated samples were destroyed and showed sparse structures. The inhibitory effect of TASA on* S. epidermidis* isolate 30 biofilm was better than the other two antibiotics; bacteria were scattered to small clumps after a treatment of TASA (Figure 3(b)). It was found that only monolayer bacteria assembled together and their structure was sparse in CIP treated biofilm (Figure 3(c)). The inhibitory effect of ERY was less than other two drugs in comparison with ATCC12228 and ATCC35984, and* S. epidermidis* isolate 30 showed a drug resistance to ERY (Figure 3(d)). According to Performance Standards for Antimicrobial Susceptibility Testing published by Clinical and Laboratory Standards Institute, Version, 2014 [24], MIC = 1–4 *μ*g/mL of* Staphylococcus* was referred to as intermediary between sensitive and resistance to CIP, MIC ≥ 4 *μ*g/mL of* Staphylococcus* was suggested to be resistant to CIP, and MIC ≤ 1 *μ*g/mL of* Staphylococcus* was suggested to be sensitive to CIP; MIC ≤ 0.5 *μ*g/mL of* Staphylococcus* was determined as sensitive to ERY; 1 ≤ MIC ≤ 4 *μ*g/mL of* Staphylococcus* was referred to as intermediary between sensitive and resistance to ERY, while MIC ≥ 8 *μ*g/mL of* Staphylococcus* was suggested to be resistant to ERY. Herein, the* S. epidermidis* isolate 30 field strain was determined as an intermediary resistance to ERY in this study. It was obvious that all of the TASA, ERY, and CIP could inhibit the biofilm formation of* S. epidermidis*.

### 3.6. Effects of TASA, ERY, and CIP on the Morphological Structure of Biofilm of* S. epidermidis* Isolate 30 Examined by CLSM

**Figure 4:**
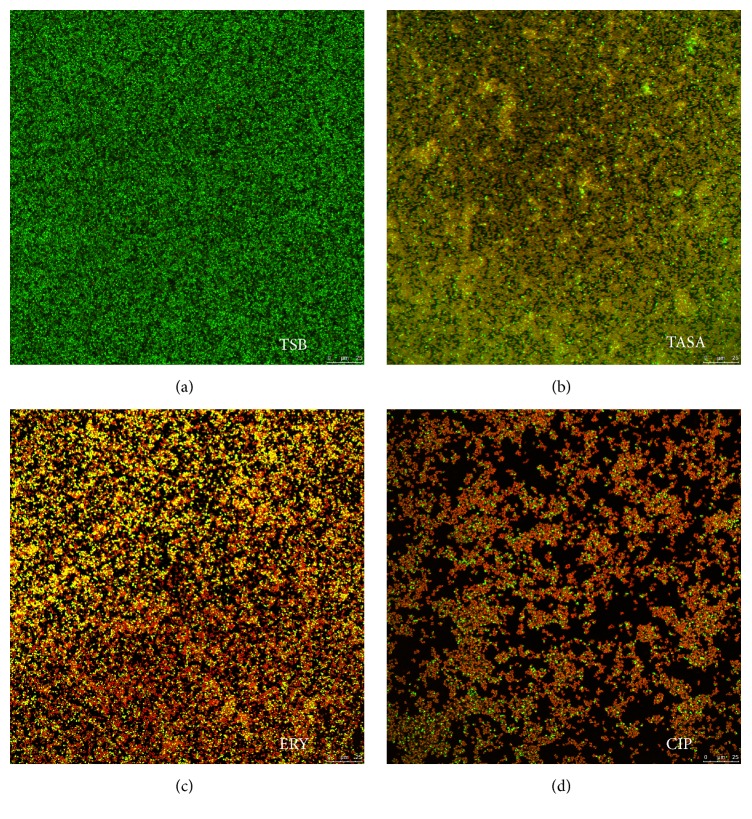
The effects of antimicrobial agents on the biofilm formation in* S. epidermidis* determined by a CLSM analysis. The* S. epidermidis* isolate 30 cells were treated with TSB control (a), 10 mg/mL of TASA (b), 500 mg/L of CIP (c), and 500 mg/L of ERY (d), and the morphological structure of biofilm was visualized under a CLSM.

Images of the biofilm of* S. epidermidis* isolate 30 treated with TASA for 24 h were compared with those treated with CIP and ERY (Figures 4(a)–4(d)). The green signal represented live bacteria that were stained by SYTO9; the red signal represented dead bacteria that were dyed by PI; the orange part in the picture was caused by the overlay of live bacteria and dead ones. ISA parameters of biofilm treated with TASA, CIP, and EYR were listed in Tables 3, 4, and 5, respectively. Results showed that biofilm in control group formed a dense net structure and had no intermediate spaces, in which bacteria were distributed into clumps (Figure 4(a)). In 10 mg/mL TASA treatment group, the number of live bacteria was significantly decreased, and the majority of areas were impaired by fluorochrome and with a significantly increased orange signal. The structure of biofilm was thin and sparse (Figure 4(b)). ISA software analysis further revealed that the thickness, biomass, average diffusion distance, and TE of biofilm were all decreased, except an increased areal porosity, when 10 mg/mL TASA treatment was compared with the control (Table 3).

Compared to the control group, results shown in Figure 4(d) and Table 4 indicated that both 62.5 *μ*g/mL and 500 *μ*g/mL CIP could inhibit the biofilm formation of* S. epidermidis*, and a higher concentration of CIP could be a better choice. The structure of biofilm was thin and sparse and its thickness became thinner when treated with 500 *μ*g/mL of CIP. The number of dead bacteria was more than that of live ones, resulting in a significantly enhanced intensity of orange (Figure 4(b)). Biovolume, textual entropy, and average diffusion distance were decreased while the areal porosity number increased, showing that the drug resistance of bacteria was reduced (Table 4). These results shown in Figure 4(c) and Table 5 indicated that at 62.5 *μ*g/mL ERY could decrease the biomass and thickness of biofilm, but it could not effectively inhibit the biofilm formation, compared with the control group. At 500 *μ*g/mL, CIP exhibited a better effect on the inhibition of biofilm formation, which could further reduce the thickness of biofilm. Biovolume, textual entropy, and average diffusion distance were decreased and areal porosity was increased. However, the drugs do not completely destroy the biofilm structure.

## 4. Discussion

Compared to planktonic bacteria, biofilm bacteria are more resistant to antibiotics. In this regard, bacteria can develop drug resistance or even multiresistance along with a long-term application of antibiotics, in particular biofilm bacteria that are even more difficult to be eliminated with conventional antibiotics, suggesting there is a need to develop novel agents to control the bacterial biofilm [4, 25]. Nowadays, the active components isolated from medicinal plants have intensively been studied for their antibacterial effects against planktonic bacteria. More importantly, some plants such as salicylic acid, houttnin, and berberine have demonstrated an ability to eliminate biofilm bacteria [26, 27]. Additionally, some traditional Chinese medicines have been reported to be able to prevent the formation of biofilm in some pathogens including* Pseudomonas aeruginosa*,* Streptococcus pyogenes*,* Streptococcus mutans*, and* Staphylococcus aureus* [28]. TASA is a type of alkaloid mixture that has also been demonstrated to have broad antibacterial activities [15].

In this study, MICs of TASA, CIP, and ERY were tested on a* S. epidermidis* field strain and* S. epidermidis* isolate 30, using a broth dilution assay. The MIC of TASA was higher than those of CIP and ERY in planktonic bacteria, despite the fact that MIC value of TASA in this study was lower than that reported in a study by Guan et al., in which the authors showed that a sub-MIC of TASA had an inhibitory effect on* S. epidermidis* biofilm formation according to SMIC value [20]. These results indicated that once the bacteria biofilm was formed, the bacterial resistance to antibiotics was significantly increased. However, differed from ERY and CIP, sub-MIC of TASA showed a capacity to inhibit biofilm bacteria significantly. The reason for this phenomenon might be attributed to the fact that biofilm bacteria were in a dormant and low metabolic state; thus ERY and CIP were difficult to play a role in inhibiting biofilm bacteria. In addition, bacteria biofilm can act as physical barriers; antibiotics thus are difficult to permeate into biofilm. Our study showed that antibiotics (ERY and CIP) had better inhibitory effect on planktonic bacteria; in terms of biofilm bacteria, traditional Chinese medicine TASA may have more inhibitory effect relative to some common used antibiotics. This finding was in line with studies of several other herbal compounds. For example, a study performed by Amaya et al. showed that garlicin could inhibit* Pseudomonas aeruginosa* adhesion through a mechanism of suppressing the expression of virulent factors related to regulation of bacterial quorum sensing [29]; similarly, another study by Shuang et al. also found that baicalin could inhibit* Enterococcus* biofilm by inhibiting the adhesive capacity in* Enterococcus* [30]. Our previous studies on TASA also revealed that it could reduce the activity of Extended Spectrum Beta-Lactamases (ESBLs) and reverse the antibiotic resistance to cefotaxime and ceftazidime in* Escherichia coli* [17].

In this study, we attempted to investigate the effect of TASA on biofilm formation of* S. epidermidis*. To this end, an XTT assay was employed to test the effect of TASA, CIP, and ERY on biofilm in different phases of culture at different concentrations. The results showed that these three agents had a clear dose-dependent effect on biofilm formation in the initial stage and their bactericidal ability was enhanced with an increasing drug concentration. However, with the biofilm growth, the antibiotics became ineffective but even promoted the biofilm formation at a certain extent. This phenomenon was consistent with a previous study that ERY could penetrate into the* S. epidermidis* biofilm but failed to kill the cells thoroughly [31]. The lower growth rate of the cells within biofilm could therefore help to decrease susceptibility to ERY; accordingly the susceptibility of biofilm bacteria to this agent was dramatically decreased with biofilm growth [31]. Several lines of evidence also showed that CIP was difficult to penetrate biofilm because of alginate, a component of biofilm. Therefore, CIP was more difficult to penetrate biofilm along with the thickness of biofilm, and previous studies about biofilm growth curve had showed that the growth of biofilm was increased with time [32]. As a result, above viewpoints could explain why ERY and CIP exhibited a less effective inhibition on biofilm bacteria as compared with the TASA at late stages of biofilm formation after 48 h and 72 h of cultures.

In order to further confirm their effects on biofilm structure, CLSM was applied for acquiring images, and the ISA software was used to analyze the biofilm structure of* S. epidermidis* isolate 30. The images and data showed that the biofilm could be partially eliminated after they were treated by TASA, ERY, and CIP alone. These results indicated that all experimental agents can inhibit biofilm bacteria, although ERY and CIP at respective 125 times and 1000 times of their MICs on planktonic cells but TASA showed an ability to inhibit biofilm at a sub-MIC concentration of planktonic state. Thus, TASA displayed the most effective effect on inhibition of biofilm formation among these three agents. This view was consistent with previous findings in* Pseudomonas aeruginosa* and* Candida albicans* [33, 34], except that the* S. epidermidis* isolate 30 used in this study was isolate from a mastitis cow, suggesting a clinical relevance of this study, but this isolate may not be a typical isolate that is able to represent all* S. epidermidis* species.

An infection of* S. epidermidis* is a main cause of bovine subclinical mastitis that is hard to be eliminated.* S. epidermidis* is easily recurrent and able to attack the host after cows are breasted and subsequently may cause a significantly economic loss in diary industry. Biofilm formation is a key reason for the difficulty of pathogen elimination and recurrent infections. However, when the disease was treated with antibiotics for a long time, the persistence of antibiotic residue is a harmful issue in public health. The Chinese medicinal herbs are less toxic, along with the richness of availability, which may be better alternative agents for the preventing and restraining bovine subclinical mastitis in veterinary clinical settings [35]. Indeed, the results presented in this study showed that TASA was able to eliminate biofilm bacteria, especially at late stages of biofilm formation.

## 5. Conclusions

This study provides a conceivable evidence that the compound of Chinese herb, total alkaloids of* Sophora alopecuroides* (TASA), has an inhibitory effect on biofilm formation of field* S. epidermidis* isolated from cow with mastitis. Moreover, TASA has an advantage over ERY and CIP in inhibition of biofilm-producing bacteria, which may provide a solution for the clinical treatment of infection related to infections of* S. epidermidis*.

## Figures and Tables

**Table 1:** The antibacterial activity of TASA and ERY alone or in combination^*∗*^.

Bacterial strains	Individual MIC		Combined MIC			
	TASA (mg/mL)	ERY (*μ*g/mL)	TASA (mg/mL)	ERY (*μ*g/mL)	FIC	Effect
ATCC12228	10	1	5	0.125	0.625	ADD
ATCC35984	10	2	12.5	0.5	1.25	IND
*S. epidermidis* 30	20	4	15	2	1.0	IND

*∗*: results represented the means of three independent experiments. ADD: additive effect; IND: no interaction.

**Table 2:** The antibacterial activity and combined effects of TASA and CIP alone or in combination^*∗*^.

Bacterial strains	Individual MIC		Combined MIC			
	TASA (mg/mL)	CIP (*μ*g/mL)	TASA (mg/mL)	CIP (*μ*g/mL)	FIC	Effect
ATCC12228	10	0.25	5	0.25	1.00	ADD
ATCC35984	10	0.25	12.5	0.5	2	IND
*S. epidermidis* 30	20	0.5	7.5	0.5	1.00	ADD

*∗*: results represented the means of three independent experiments. ADD: additive effect; IND: no interaction.

**Table 3:** Parameterized analysis of biofilm structure treated with TASA^*∗*^.

Samples	BF thickness	BV	AP	ADD	TE
Control	19.64 ± 0.5	3931422 ± 19549.78	0.75 ± 0.08	1.32 ± 0.13	6.89 ± 0.73
TASA (1.25 mg/mL)^▲^	12.59 ± 0.50^**∗**^	2879970 ± 3210.27^**∗∗**^	0.79 ± 0.071	1.37 ± 0.26	5.38 ± 0.71^**∗**^
TASA (10 mg/mL)	4.28 ± 0.2^**∗∗**▲▲^	564108 ± 3130.5^**∗∗**▲▲^	0.88 ± 0.01^**∗**^	1.10 ± 0.01	4.15 ± 0.43^**∗**^

*∗*: the biofilm structure was analyzed using ISA software. Compared to the control group, ^*∗*^ *p* < 0.05 and ^*∗∗*^ *p* < 0.01; compared the 1.25 mg/mL TASA group, ^▲^ *p* < 0.05 and ^▲▲^ *p* < 0.01.

**Table 4:** Parameterized analysis of BF structure treated with CIP^*∗*^.

Samples	BF thickness	BV	AP	ADD	TE
Control	19.64 ± 0.50	3931422 ± 19549.78	0.75 ± 0.08	1.32 ± 0.13	6.89 ± 0.74
CIP (62.5 mg/L)^▲^	16.62 ± 0.50^**∗**^	1843982 ± 10367.5^**∗∗**^	0.78 ± 0.05	1.31 ± 0.42	4.89 ± 1.45^**∗∗**^
CIP (500 mg/L)	8.13 ± 0.07^**∗∗**▲▲^	575536 ± 11506.5^**∗∗**▲▲^	0.91 ± 0.02^**∗∗**^	1.09 ± 0.02	3.69 ± 0.57^**∗∗**^

*∗*: the biofilm structure was analyzed using ISA software. Compared to the control group, ^*∗*^ *p* < 0.05 and ^*∗∗*^ *p* < 0.01; compared the 62.5 mg/L CIP group, ^▲^ *p* < 0.05 and ^▲▲^ *p* < 0.01.

**Table 5:** Parameterized analysis of BF structure treated with ERY^*∗*^.

Samples	BF thickness	BV	AP	ADD	TE
Control	19.64 ± 0.50	3931422 ± 19549.78	0.75 ± 0.08	1.32 ± 0.13	6.89 ± 0.74
ERY (62.5 mg/L)^▲^	16.11 ± 0.67^**∗**^	2887001 ± 1780^**∗∗**^	0.83 ± 0.06^**∗**^	2.46 ± 0.47	5.08 ± 1.32^**∗**^
ERY (500 mg/L)	12.59 ± 0.34^**∗∗**^	2007005 ± 4782.67^*∗∗*▲▲^	0.86 ± 0.10^**∗**^	1.23 ± 0.02	4.97 ± 0.48^**∗**^

*∗*: the biofilm structure was analyzed using ISA software. Compared to the control group, ^*∗*^ *p* < 0.05 and ^*∗∗*^ *p* < 0.01; compared the 62.5 mg/L ERY group, ^▲^ *p* < 0.05 and ^▲▲^ *p* < 0.01.

## Abbreviations

*S. epidermidis*: * Staphylococcus epidermidis*
TASA: Total alkaloids of* Sophora alopecuroides*
MIC: Minimal inhibitory concentration
SMIC: Subminimal inhibitory concentration
CIP: Ciprofloxacin
ERY: Erythromycin
BF: Biofilm
XTT: Sodium 3′-[1-[(phenylamino)-carbony]-3,4-tetrazolium]-bis(4-methoxy-6-nitro) benzene-sulfonic acid hydrate.
